# Compact vision models match domain-specific foundation models for several retinal imaging classification tasks: A systematic benchmark

**DOI:** 10.1371/journal.pone.0356202

**Published:** 2026-08-18

**Authors:** Dávid Isztl, Tahm Spitznagel, Gábor Márk Somfai, Rui Santos

**Affiliations:** 1 Stadtspital Zurich, Department of Ophthalmology, Zurich, Switzerland; 2 Spross Research Institute, Zurich, Switzerland; National Taichung University of Science and Technology, TAIWAN

## Abstract

Large domain-specific foundation models have been widely adopted for retinal image analysis, yet systematic evidence for their advantage over compact general-purpose architectures remains scarce. We benchmarked nine model configurations spanning 22.8M to 303M parameters (vision transformers, hierarchical Swin Transformers, ConvNeXt, and the domain-specific RETFound models) across four tasks: OCT 8-class disease classification, and three fundus photography tasks (DME severity, glaucoma detection, and DR severity grading). All models were evaluated under identical training conditions, with both pretrained (on natural-domain image datasets) and from-scratch initializations compared using Mann-Whitney U tests. Pretraining improved accuracy by 5.18–18.41 percentage points across all tasks (p < 0.05 throughout), with larger benefits for CFP modalities and harder tasks. Compact hierarchical models (27–29M parameters) matched or exceeded larger architectures on three of four tasks. For instance, the SwinV2-tiny architecture ranked first on OCT, DME, and GL classification. The domain-specific RETFound model (303M) achieved the highest accuracy only on the most challenging task (DR severity grading, where the most severe class is underrepresented at 8% of images), where it outperformed the best compact model by 1.54 percentage points. These results indicate that compact general-purpose models may be sufficient for most retinal classification benchmarks, and that domain-specific foundation models may add higher value mainly for severity grading tasks with skewed class distributions.

## Introduction

Retinal imaging provides non-invasive access to vascular and neural tissue, enabling detection and monitoring of sight-threatening diseases including diabetic retinopathy, macular edema, and glaucoma. With over 500 million people affected by diabetes worldwide [1] and glaucoma representing the leading cause of irreversible blindness [2], automated classification systems offer genuine potential for scaling screening programs in resource-limited settings. Beyond ocular pathology, the emerging field of oculomics further expands the utility of retinal imaging toward systemic disease screening [3–5].

Deep learning has achieved strong performance across a broad range of retinal classification tasks, with convolutional networks and vision transformers establishing high baselines on multiple benchmarks [6]. A recent trend has been the development of large domain-specific foundation models, such as RETFound [7,8], a 303M-parameter model self-supervised on large-scale retinal image collections. The rationale being that natural-image features do not transfer well to specialized retinal imaging. This has motivated substantial computational investments in domain-specific pretraining pipelines.

However, systematic comparisons of these large, specialized models against compact general-purpose architectures across multiple tasks and imaging modalities remain scarce [9–11]. Most prior work evaluates a single architecture on a single dataset, making it difficult to draw generalizable conclusions about when scale or domain specificity is needed. Thus, key practical questions remain unanswered: Do compact 27–29M parameter architectures suffice for most retinal imaging tasks? Under what conditions (imaging modality, class balance, task difficulty) does domain-specific pretraining provide measurable advantages over standard ImageNet [12] initialization?

We address these questions with a controlled benchmark across four retinal classification tasks and two imaging modalities (OCT and CFP). We evaluate nine backbone configurations under identical training conditions, comparing pretrained and from-scratch initialization. Our contribution is empirical rather than architectural: we provide a controlled comparison of compact and domain-specific foundation models under identical training conditions across multiple tasks and modalities, as a reference for practitioners choosing between compact general-purpose models and larger domain-specific alternatives for the future development of vision-based Foundation Models.

## Methods

### Datasets

We evaluated models on four classification tasks across two retinal imaging modalities. Table 1 summarizes dataset sources, sizes, class counts, and key characteristics.

**Table 1:** Retinal imaging datasets used in this study. Task difficulty increases from OCT (eight balanced classes) to DR (five severity grades, severely imbalanced). Split ratios are provided or stratified as indicated.

Task	Source(s)	Images	Classes	Split	Key characteristics
OCT	Retinal OCT C8 [13]	24,000	8	Provided	Balanced; 8 disease categories (AMD, CNV, CSR, DME, DR, Drusen, MH, Normal); 2,300/350/350 per class
DME	IDRiD [14] + Messidor-2 [15]	2,264	3	70/15/15	Small combined dataset; grades 0–2 by hard-exudate distance from macula
GL	AIROGS [16] + PAPILA [17]	114,381	3	70/15/15	Large, combined screening + diagnostic dataset; unified label space {0,1,2}
DR	EAM [18]	143,669	5	80/10/10	Severe class imbalance (Severe NPDR: 8.22%); ordinal ICDR scale 0–4

OCT (Retinal OCT C8 [13], https://www.kaggle.com/datasets/obulisainaren/retinal-oct-c8): 24,000 images across 8 balanced disease categories (AMD, CNV, CSR, DME, DR, Drusen, MH, Normal), with pre-defined splits of 2,300 training, 350 validation, and 350 test images per class. DME (IDRiD [14] + Messidor-2 [15], https://zenodo.org/records/17219542 and https://www.kaggle.com/datasets/mariaherrerot/messidor2preprocess respectively): 2,264 combined fundus images with 3-class DME severity labels (0: no DME, 1: non-center-involving, 2: center-involving), stratified split 70/15/15. GL (AIROGS [16] + PAPILA [17], https://airogs.grand-challenge.org/data-and-challenge/ and https://www.kaggle.com/datasets/orvile/papila-retinal-fundus-images respectively): 114,381 fundus images combined from a population screening dataset (AIROGS, binary labels) and a diagnostic dataset (PAPILA, 3-class labels), mapped to a unified label space {0, 1, 2}, stratified split 70/15/15. DR (EAM unified dataset [18], https://www.kaggle.com/datasets/ascanipek/eyepacs-aptos-messidor-diabetic-retinopathy): 143,669 fundus images on the 5-class International Clinical Diabetic Retinopathy (ICDR) ordinal scale (0: No DR to 4: Proliferative DR), with severe class imbalance (Severe NPDR class: 8.22%), stratified split 80/10/10.

*This study used exclusively publicly available, de-identified datasets. No ethics approval was required.*

### Backbone architectures and initialization strategies

Table 2 lists all nine backbone architectures evaluated. The selection spans supervised and self-supervised vision transformers, hierarchical Swin Transformers, a modern convolutional architecture (ConvNeXtV2), and domain-specific retinal foundation models (RETFound). All pretrained weights were sourced from the HuggingFace model hub.

**Table 2:** Backbone architectures evaluated. All models were fine-tuned end-to-end from pretrained weights, except where noted. Four architectures (ViT-base, Swin-tiny, SwinV2-tiny, ConvNeXtV2-tiny) were also trained from random initialization to quantify the pretraining benefit. DINOv2 variants and RETFound models were evaluated with pretrained weights only.

Family	Model*	Params
ViT [19]	ViT-base	86.6M
Self-supervised ViT [20]	DINOv2-small	22.8M
	DINOv2-small-reg	86.6M
Swin Transformer [21,22]	Swin-tiny	28.3M
	SwinV2-tiny	27.6M
ConvNeXt [23]	ConvNeXtV2-tiny	28.6M
Domain-specific (RETFound [7,8])	RETFound-MAE-OCT [7]	303M
	RETFound-MAE-CFP	303M
	RETFound-DINOv2-CFP	303M

*HuggingFace identifiers: ViT-base: google/vit-base-patch16–224; DINOv2-small: facebook/dinov2-small-imagenet1k-1-layer; DINOv2-small-reg: facebook/dinov2-with-registers-base; Swin-tiny: microsoft/swin-tiny-patch4-window7–224; SwinV2-tiny: microsoft/swinv2-tiny-patch4-window8–256; ConvNeXtV2-tiny: facebook/convnextv2-tiny-1k-224; RETFound-MAE-OCT: iszt/RETFound_mae_natureOCT; RETFound-MAE-CFP: iszt/RETFound_mae_meh; RETFound-DINOv2-CFP: iszt/RETFound_dinov2_meh.

Two initialization strategies were compared: (1) Pretrained: backbones initialized with ImageNet-1k weights (or retinal-specific weights for RETFound models), then fine-tuned end-to-end; (2) From scratch: random initialization. Four architectures (ViT-base, Swin-tiny, SwinV2-tiny, ConvNeXtV2-tiny) were evaluated under both strategies; DINOv2 variants and RETFound models were evaluated with pretrained weights only. This yielded 13 configurations for OCT and 12 for each CFP task.

### Training procedure

To ensure fair comparison, all models were trained on a unified platform and under similar conditions. We used AdamW optimization [24] with cosine learning rate schedules, 10% linear warmup, weight decay 0.05, and gradient clipping (max norm 1.0). Learning rates were selected per architecture and initialization strategy following established practices for fine-tuning vision transformers [25]. For pretrained models, learning rates were: ViT-base 3 × 10 ^-4^, DINOv2-small 3 × 10 ^-4^, DINOv2-with-registers 2 × 10^-4^, Swin-tiny 5 × 10^-4^, SwinV2-tiny 4 × 10 ^-4^, ConvNeXtV2-tiny 1 × 10^-3^, and RETFound variants 3 × 10^-4^. For models trained from scratch, the learning rates were: ViT-base 5 × 10^-4^, Swin-tiny 5 × 10^-4^, SwinV2-tiny 5 × 10^-4^, and ConvNeXtV2-tiny 1 × 10^-3^ (not applicable for DINOv2 and RETFound models). Effective batch size was 256 via gradient accumulation; training ran for 100 epochs without early stopping, with the best validation accuracy reported. Data augmentation was intentionally minimal (resize, center crop, normalization) to isolate architecture and initialization effects. Cross-entropy loss was used without class weighting. All experiments used mixed precision (bfloat16), fixed random seed 42, and one NVIDIA RTX PRO 6000 GPU (Blackwell).

### Evaluation metrics and statistical analysis

We report macro-averaged metrics per model: top-1 validation accuracy, macro-averaged AUROC [26], macro-averaged F1-score [27], and Cohen’s Kappa [28]. Cohen’s Kappa adjusts agreement for chance and is particularly informative under class imbalance. Macro-averaging was chosen to give equal weight to each class regardless of support, providing a conservative estimate of performance on imbalanced tasks. Per-class metrics are not reported in the main text as we focus on cross-architecture and cross-initialization comparisons at the aggregate level; this follows the reporting standard established by recent foundation model evaluations in retinal imaging [7]. Pretrained vs. scratch differences were assessed using two-sample Mann-Whitney U tests (α = 0.05) [29]. Pareto frontier analysis [30] identified models that are optimal in the accuracy-vs-parameter-count trade-off space (no other model achieves higher accuracy with fewer parameters). Pearson correlation was used to assess the linear relationship between model size and performance metrics.

## Results

### Pretraining consistently improves performance

Pretrained models outperformed their scratch-trained counterparts on all four tasks (Table 3, p < 0.05, Mann-Whitney U). This benefit ranged from 5.18 to 18.41 percentage points and followed two consistent patterns. First, it scaled with task difficulty: the hardest task (DR severity grading, five grades, severely imbalanced) showed the largest gain (+18.41 pp), while the easiest task (OCT, 8-class balanced) showed the smallest (+5.18 pp). Second, CFP-based tasks (DME, GL, DR) consistently showed larger pretraining advantages (9.13–18.41 pp) than OCT (5.18 pp), suggesting that ImageNet’s natural image statistics transfer more readily to color fundus photography than to cross-sectional OCT imaging. Beyond accuracy, pretraining substantially reduced performance variance: scratch-trained models showed 2–4 × higher standard deviations than their pretrained counterparts across all tasks.

**Table 3:** Pretraining benefit across all four tasks. Values reported as mean ± standard deviation over all pretrained (np) and scratch-trained (ns) configurations per task. Differences are absolute percentage points in validation accuracy. All differences are statistically significant (p < 0.05, Mann-Whitney U test [29]). The pretraining advantage scales with task difficulty and is consistently larger for color fundus photography (CFP) tasks than for OCT.

Task	Modality	np / ns	Pretrained acc	Scratch acc	Gain (pp)	p-value
OCT (8-class balanced)	OCT	9 / 4	0.969 ± 0.009	0.918 ± 0.034	+5.18	0.003
GL (3-class)	CFP	8 / 4	0.907 ± 0.022	0.815 ± 0.059	+9.13	0.004
DME (3-class)	CFP	8 / 4	0.981 ± 0.021	0.874 ± 0.077	+10.70	0.022
DR (five severity grades)	CFP	8 / 4	0.671 ± 0.029	0.487 ± 0.081	+18.41	0.004

### Compact models match large models on most tasks

On three out of the four tasks (OCT, DME, GL), the best compact model (≤30M; Table 4) equaled or outperformed all larger architectures including the domain-specific 303M-parameter RETFound models. SwinV2-tiny (27.6M) ranked first on OCT, DME, and GL. On the DR task, RETFound-DINOv2-CFP (303M) achieved the highest accuracy (71.15%), with SwinV2-tiny as the runner-up (69.61%), a gap of 1.54 percentage points.

**Table 4:** Per-task best-performing model overall and best compact model (≤30M parameters, marked †). On OCT, DME, and GL the best compact model equals the best overall model. On DR the 303M domain-specific model outperforms the best compact alternative by 1.54 percentage points. Acc: top-1 validation accuracy. AUROC: macro-averaged area under the ROC curve. F1: macro-averaged F1-score.

Task	Model	Params	Acc (%)	AUROC	F1
OCT (8-class)	ConvNeXtV2-tiny^†^	28.6M	97.96	0.9988	0.9797
DME (3-class)	SwinV2-tiny^†^	27.6M	99.24	0.9997	0.9924
GL (3-class)	SwinV2-tiny^†^	27.6M	93.19	0.9806	0.8475
DR (best overall)	RETFound-DINOv2-CFP	303M	71.15	0.9323	0.6998
DR (best compact)	SwinV2-tiny^†^	27.6M	69.61	0.9202	0.7020

Table 5 shows cross-task architecture rankings. SwinV2-tiny achieved the most consistent performance (mean rank 1.50 across all four tasks). RETFound-DINOv2-CFP ranked first on all three CFP tasks when available, but this advantage was concentrated on the hardest task (DR); on DME and GL, SwinV2-tiny achieved higher accuracy. Standard ViT-base (86.6M) showed the most inconsistent performance, ranking 5th–7th across tasks. Pearson correlation between parameter counts and accuracy was non-significant on all tasks (p > 0.24 throughout), with similar results for AUROC and F1, building evidence that model scale alone does not predict performance in these benchmarks.

**Table 5:** Architecture performance consistency across all four tasks. Ranks are based on best validation accuracy within each task (rank 1 = best). Mean rank summarizes cross-task robustness. Compact hierarchical models (SwinV2-tiny, Swin-tiny, ConvNeXtV2-tiny) show the most consistent performance. RETFound-DINOv2-CFP achieves the top rank only on the most challenging task (DR); it is unavailable for OCT evaluation (trained on CFP data). DINOv2 models were evaluated with pretrained weights only and are included for reference.

Architecture	Params	OCT	DME	DR	GL	Mean rank
SwinV2-tiny	27.6M	1	1	2	2	1.50
RETFound-DINOv2-CFP	303M	–	2	1	1	1.33*
Swin-tiny	28.3M	3	8	3	3	4.25
ConvNeXtV2-tiny	28.6M	2	5	5	4	4.00
DINOv2-small-reg	86.6M	5	4	4	6	4.75
ViT-base	86.6M	7	7	7	5	6.50

*Mean computed over CFP tasks only (DME, DR, GL); RETFound-DINOv2-CFP not applicable to OCT.

### Pareto analysis: Scale does not pay off except for DR

**Fig 1:**
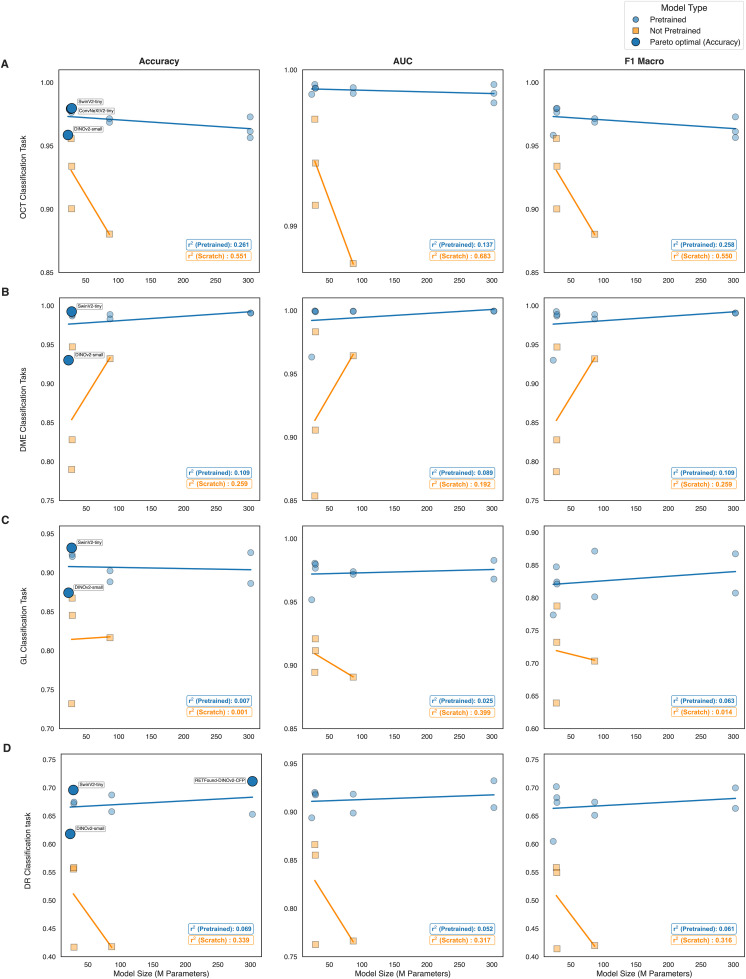
Model performance versus model size across four ophthalmic imaging classification tasks. Validation Accuracy, AUC, and F1 Macro scores are plotted against model size (in millions of parameters) for the (A) OCT, (B) DME, (C) GL, and (D) DR classification tasks. Blue circles represent pretrained models, while dark orange squares represent models trained from scratch (“Not Pretrained”). Linear regression lines illustrate the overall trend between model capacity and performance for each initialization strategy. The corresponding Pearson coefficient (r^2^) is displayed in the lower-right corner of each subplot, color-coded by pretraining status. Large, annotated markers in the Accuracy column indicate models identified as Pareto optimal based on accuracy only (i.e., models where no other model in the same pretraining class achieves both higher accuracy and a smaller parameter count); Pareto optimality was not computed for AUC or F1 Macro. Model name annotations display the specific architecture of the Pareto optimal points using concise labels. Y-axes are scaled dynamically within each dataset to highlight relative performance differences.

Pareto-optimal models (larger markers, accuracy-based) cluster in the 22.8–28.6M parameter range for OCT, DME, and GL (Fig 1A-1C). Only on the most challenging task for DR classification (Fig 1D) does a larger model (RETFound-DINOv2-CFP, 303M) appear on the Pareto frontier, where it offers a 1.54 percentage point accuracy gain over the best compact alternative (SwinV2-tiny). On OCT, DME, and GL, models above 30M are strictly dominated. Thus, higher parameter counts deliver no meaningful accuracy, AUC, or F1 benefit. Linear regression lines and low r² values per subplot further confirm that pretraining status, not model size, is the dominant predictor of performance.

### Parameter efficiency analysis

**Fig 2:**
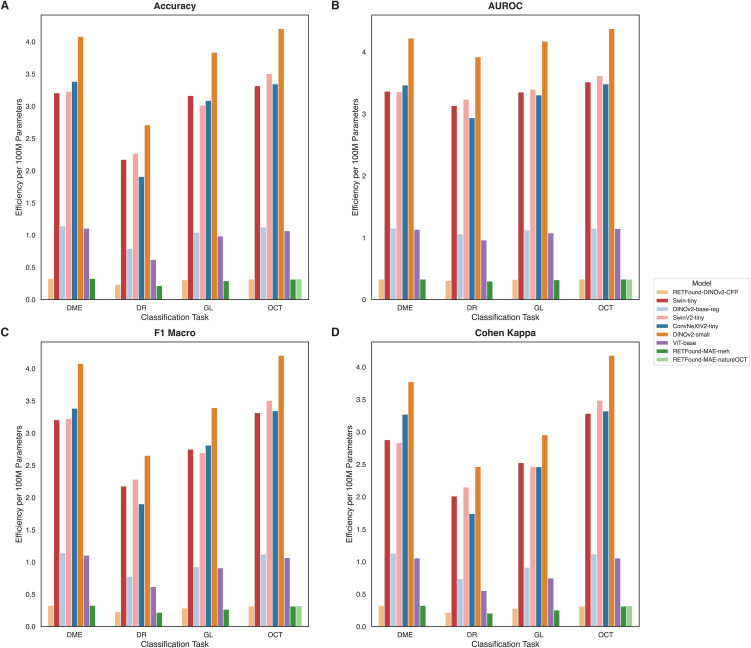
Model efficiency per 100 million parameters across four retinal imaging classification tasks. Efficiency metrics, (A) validation accuracy, (B) AUROC, (C) F1 macro, and (D) Cohen’s kappa were computed as the ratio of each metric to model size (in 100M parameters) for all evaluated models on the DME, DR, GL, and OCT classification tasks. Bars are colored by model name (see legend). Only pretrained models are evaluated. Higher values represent greater parameter efficiency, enabling direct comparison of architectures normalized by computational cost.

To contextualize the cost-to-benefit trade-off beyond the raw Pareto frontier, we computed a normalized parameter efficiency metric for each model, defined as the model’s best validation performance divided by its parameter count, expressed per 100 million parameters: Efficiency = Accuracy / Model Size (M) × 100. This yields the performance delivered per 100 million parameters, where higher values indicate greater parameter efficiency. Fig 2 visualizes this metric across all four tasks and four performance measures (Accuracy, AUROC, F1 Macro, Cohen’s Kappa). DINOv2-small (22.8M) achieves the highest parameter efficiency across all four tasks and all four metrics, with accuracy-based efficiency values of approximately 4.2–4.3 per 100M parameters on DME, glaucoma, and OCT, and approximately 2.5 on the more challenging DR task. A second tier of compact models, SwinV2-tiny (27.6M), Swin-tiny (28.3M), and ConvNeXtV2-tiny (28.6M), achieves efficiency values in the range of 1.9–3.5 per 100M parameters depending on task and metric. In contrast, the three 303M-parameter RETFound variants exhibit the lowest efficiency (approximately 0.25–0.35 per 100M parameters for accuracy), an order of magnitude below the compact models, because their large parameter count dramatically dilutes the per-parameter performance gain. ViT-base (86.6M) and DINOv2-base-reg (86.6M) fall in an intermediate range (approximately 0.6–1.5 per 100M parameters). The relative ranking of models is remarkably consistent across all four metrics (Accuracy, AUROC, F1 Macro, Cohen’s Kappa), reinforcing the robustness of the comparison. Additionally, task difficulty modulates absolute efficiency: the DR task, the only 5-class ordinal grading problem with severe class imbalance, yields the lowest efficiency values across all models, while OCT generally yields the highest. This analysis underscores a key finding: for three of four retinal imaging tasks, compact pretrained models deliver substantially more performance per unit of model capacity than domain-specific foundation models. The per-parameter advantage of compact models is not merely marginal but spans an order of magnitude. Foundation model scale appears to pay off only in absolute terms on the most challenging task (DR), and even there, the efficiency cost remains high. These findings support a task-dependent rather than universal recommendation for domain-specific foundation models.

## Discussion

### Pretraining is necessary; scale is not

The most consistent finding across all four tasks is that transfer learning substantially benefits retinal disease classification, whether from ImageNet or retinal-specific pretraining. Scratch-trained models not only underperformed on average but also showed much higher performance variance, suggesting that random initialization leads to more variable and less reliable convergence.

The finding that compact 27–29M parameter models match or outperform larger models on three of four tasks is practically relevant: several retinal classification tasks may be addressed without large-scale foundation model infrastructure. SwinV2-tiny, with 27.6M parameters and ImageNet initialization, achieved first place on three tasks and second place on the fourth, making it the most consistently high-performing architecture in this comparison. Its hierarchical design, which extracts features at multiple spatial scales, appears well-suited to the diverse feature scales relevant in retinal pathology, ranging from small microaneurysms to large structural patterns such as optic disc morphology.

### Domain-specific pretraining is task-selective

RETFound (303M) outperformed compact ImageNet-pretrained models on one task only: DR severity grading across five imbalanced classes. On DME, GL, and OCT, compact ImageNet-pretrained models performed equivalently or better. Domain-specific pretraining appears to help when the task demands subtle discrimination between severity grades that are visually similar and sparsely represented.

The DR result itself warrants careful interpretation. The 1.54 percentage point accuracy advantage of RETFound on DR severity grading corresponds to 11x increase in parameter count. To contextualize this trade-off clinically, we may consider two dimensions: absolute performance and operational impact. Regarding absolute performance, all models on DR remain substantially below ceiling (best accuracy 0.7115 for RETFound), reflecting the inherent difficulty of 5-class ordinal grading under severe class imbalance. As such, what constitutes a clinically meaningful accuracy threshold depends on the specific application. Considering operational impact, for each 1,000 screened patients at base rate ~5% referable DR, the 1.54 pp accuracy gain corresponds to ~15 additional correctly classified patients. Whether this justifies the 11x increase in computational cost depends on the volume of the screening context.

Further, mechanistic understanding of RETFound’s DR advantage may benefit from future work using attention rollout, Grad-CAM, or representation similarity analysis comparing feature spaces of compact models and RETFound on DR images. We hypothesize that RETFound’s retinal-specific pretraining yields representations more sensitive to fine pathological features (microaneurysms, small hemorrhages, IRMA) that are diagnostic of severe DR but underrepresented in ImageNet. Testing this hypothesis is an important direction for follow-up work and would help establish whether the 1.54 pp advantage reflects qualitatively different feature learning or simply larger model capacity.

### Interpreting high accuracy on OCT and DME

The near-ceiling accuracy on OCT (97.96%) and DME (99.24%) likely reflects the nature of these benchmarks, not model overfitting. The OCT C8 dataset is balanced across 8 categories with clear inter-class visual differences (e.g., retinal layer disruptions in AMD vs. fluid-filled spaces in CNV), and the provided splits maintain this balance. The DME dataset, while small (2,264 images), presents a 3-class severity problem defined by hard exudate proximity to the macula, a relatively unambiguous visual criterion at the extremes of the severity spectrum. This, high accuracy on well-structured, visually distinct benchmarks is expected. The contribution of this research lies in demonstrating that this performance is achievable with compact general-purpose architectures rather than large, specialized models.

Nonetheless, these benchmark results should not be extrapolated to real-world screening contexts without further validation. Dataset-level performance on curated splits may not reflect clinical deployment, where image quality variation, acquisition device differences, and demographic diversity can substantially degrade model performance. External validation studies on independent cohorts would be required before drawing clinical conclusions.

### Model efficiency

Our parameter efficiency analysis (Fig 2) complements the Pareto frontier by quantifying how much performance each architecture delivers per unit of model capacity. The compact DINOv2-small consistently achieves the highest efficiency per 100M parameters across all tasks and metrics, with values up to 10 × higher than the RETFound variants. This finding has direct practical implications: in resource-constrained deployment settings, such as edge inference, mobile screening, or institutions with limited GPU infrastructure, compact pretrained models offer equivalent or near-equivalent clinical utility at a fraction of the computational footprint. Furthermore, pretraining itself dramatically improves parameter efficiency: for the same architecture, pretrained versions consistently outperform their scratch-trained counterparts on a per-parameter basis (e.g., SwinV2-tiny on glaucoma: pretrained accuracy efficiency ≈ 3.38 vs. scratch ≈ 2.65 per 100M parameters), reinforcing that the combination of compact architecture and effective pretraining is the most efficient pathway to strong retinal imaging performance. While a comprehensive deployment cost analysis would ideally also include training time, inference latency, and GPU memory consumption, parameter count serves as a well-established proxy for these costs, as it scales approximately with FLOPs and memory for transformer-based architectures within the same model family. We acknowledge this as a limitation and note that direct hardware profiling across architectures would further strengthen the deployment-cost argument.

### Limitations

This study has several limitations. First, there is no external validation cohort. Each task was evaluated on a single dataset or dataset combination (OCT C8, IDRiD+Messidor-2, AIROGS+PAPILA, EAM). Generalizability to other data sources, imaging devices, geographic populations, and acquisition protocols was not assessed. Conclusions about cross-task architecture rankings and pretraining benefits apply to these specific datasets and may not transfer unchanged to independent cohorts.

Second, no feature visualization or mechanistic interpretability analysis was performed. We did not perform attention map visualization, class activation mapping, or representation similarity analysis to explain why RETFound outperforms compact alternatives on DR or why hierarchical transformers consistently outperform standard ViTs. Such analyses are necessary to establish mechanistic understanding of when and why domain-specific pretraining helps. They were not included here due to scope, but they represent an important direction for future work, particularly to test the hypothesis that RETFound’s advantage on DR stems from learned representations of fine-grained pathological features such as microaneurysms.

Third, no calibration, uncertainty quantification, or failure mode analysis was performed. Our evaluation focused on classification accuracy and related metrics. Calibration uncertainty estimation, and systematic characterization of failure modes were not assessed. These are important complementary perspectives, particularly for clinical applications where knowing when a model is uncertain is as important as its average accuracy.

Fourth, demographic subgroup analysis was not performed. Algorithmic fairness across demographic subgroups (age, sex, ethnicity, disease severity) was not evaluated. Recent work has highlighted that retinal imaging models can show performance disparities across demographic groups, and we did not assess whether the architecture and pretraining choices studied here affect performance differentially across such subgroups. Multi-site datasets with demographic metadata would be required for such analysis.

Fifth, we used unweighted cross-entropy loss without task-specific adaptations (e.g., ordinal regression losses, focal loss for class imbalance), which may partially explain the lower absolute performance on the DR task. While this choice ensures fair cross-model comparison, it may underestimate achievable performance on imbalanced tasks, particularly for underrepresented severe classes.

Finally, the computational efficiency was not directly measured. We did not measure training time, inference latency, GPU memory consumption, or energy requirements across architectures. While theoretical parameter counts and FLOPs provide partial guidance, practical deployment characteristics depend on hardware, batching, and software stack. We discuss theoretical efficiency via parameter efficiency (accuracy per 100M parameters, Fig 2) but acknowledge that empirical measurements on representative hardware would strengthen the deployment guidance.

## Conclusion

We benchmarked nine model configurations (22.8M–303M parameters) across four retinal classification tasks under controlled conditions. Three findings stand out.

First, pretraining consistently outperforms random initialization, with accuracy gains of 5.18–18.41 percentage points (p < 0.05 on all tasks). The benefit is found to be larger for CFP-based tasks than for OCT, and scales with task difficulty.

Second, compact hierarchical models in the 27–29M parameter range deliver competitive or superior performance on most tasks. SwinV2-tiny (27.6M, ImageNet pretrained) ranked first on three of four tasks and second on the fourth, while models with 86.6M or 303M parameters did not provide proportional accuracy gains.

Third, domain-specific pretraining (RETFound, 303M) provides a measurable advantage only on the most challenging task (DR severity grading, five grades, severely imbalanced), where it outperformed the best compact model by 1.54 percentage points. On the remaining three tasks, ImageNet-pretrained compact models sufficed.

Thus, for most retinal classification tasks tested, compact ImageNet-pretrained models are a practical default. Large domain-specific foundation models are better justified when the task involves severity grading with skewed class distributions. Further validation on heterogeneous, multi-site datasets is needed before these findings can inform clinical deployment decisions.
