# Supplementary material for: Compact vision models match domain-specific foundation models for several retinal imaging classification tasks: A systematic benchmark
Source: PLoS One. 2026 Aug 18;21(8):e0356202. doi: 10.1371/journal.pone.0356202 (PMC13484977; doi:10.1371/journal.pone.0356202)
Supplement: Supplementary file 1 [file pone.0356202.s002.pdf]

Stadtspital Zürich

Werner H. Spross
Stiftung zur Förderung
der Augenheilkunde

Stadtspital Zürich
Augenklinik
Birmensdorferstrasse 497, 8063 Zürich
stadtspital.ch

Sekretariat
T +41 44 416 42 42, F +41 44 416 42 44
augenklinik@stadtspital.ch

Chefarzt
Prof. Dr. med. Matthias Becker

25th June 2026

To the editor Xin Sun (PhD)

PLOS ONE

Public Library of Science

Rebuttal Letter to reviewers for manuscript: *"Compact vision models match domain-specific foundation models for several retinal imaging classification tasks: A systematic benchmark"*

We thank the editor and reviewers for their thoughtful and constructive comments, which have substantially strengthened the manuscript. Below, we address each point in detail.
All changes are highlighted in the marked-up manuscript.

Journal Requirements

Requirement 1: PLOS ONE style requirements. We have reviewed and confirmed that the manuscript conforms to PLOS ONE formatting guidelines, including file naming conventions, section structure, and reference formatting.

Requirement 2: We have added a "Data availability" statement to the manuscript specifying dataset access links, and code will be deposited at the time of acceptance.

Requirement 3: Ethics statement location. We confirm that the ethics statement has been moved to the Methods section as a final subsection ("This study used exclusively publicly available,

Stadtspital Zürich
Augenklinik
Birmensdorferstrasse 497, 8063 Zürich
stadtspital.ch

Sekretariat
T +41 44 416 42 42, F +41 44 416 42 44
augenklinik@stadtspital.ch

Chefarzt
Prof. Dr. med. Matthias Becker

de-identified datasets. No ethics approval was required.") and has been removed from any other section.

Requirement 4: Funder role statement. We have updated the Funding section to read: "This work was supported by the Werner H. Spross-Stiftung. The funders had no role in study design, data collection and analysis, decision to publish, or preparation of the manuscript."
Requirement 5: Reviewer-recommended citations. We have reviewed the publications suggested by Reviewer 2 (Point 8) and have addressed this in our response below (see Reviewer 2, Point 8).

Answers to Reviewer 1

Comment 1: "No architecture novelty (the authors propose no new framework)."

We thank the reviewer for this comment and agree that this study does not propose a novel architecture. We respectfully clarify that our manuscript is an empirical benchmarking study, not a methods or architecture-development paper. Its scientific contribution lies in providing the first controlled, multi-task, multi-modal comparison of compact general-purpose models against domain-specific foundation models (RETFound) under identical training conditions. This is a distinct and valuable contribution, as the field currently lacks systematic evidence about *when* domain-specific scale is justified versus when compact, off-the-shelf models suffice. We have added an explicit statement in the Introduction clarifying this scope:
"Our contribution is empirical rather than architectural: we provide a controlled comparison of compact and domain-specific foundation models under identical training conditions across multiple tasks and modalities, as a reference for practitioners choosing between compact

Stadtspital Zürich

Werner H. Spross
Stiftung zur Förderung
der Augenheilkunde

Stadtspital Zürich
Augenklinik
Birmensdorferstrasse 497, 8063 Zürich
stadtspital.ch

Sekretariat
T +41 44 416 42 42, F +41 44 416 42 44
augenklinik@stadtspital.ch

Chefarzt
Prof. Dr. med. Matthias Becker

general-purpose models and larger domain-specific alternatives for the future development of vision-based Foundation Models.*"*

We believe the value of systematic benchmarking is well-established in the machine learning community. Our study serves the same purpose for retinal imaging specifically.

Comment 2: "There are not enough figures to show the difference between models' performance."

We agree that the original single-figure submission was insufficient. We have substantially expanded the visual presentation:

- Figure 1 has been completely redesigned. It now comprises a multi-panel figure showing validation Accuracy, AUROC, and F1 Macro for all four tasks (OCT, DME, GL, DR) plotted against model size, with pretrained and scratch-trained models shown separately, Pareto-optimal models highlighted with annotated markers, and per-subplot linear regression lines with r^2 values. This provides a comprehensive visual comparison across all models, tasks, and metrics simultaneously.
- Figure 2 is newly added, presenting a 4-panel bar chart showing parameter efficiency (performance per 100M parameters) across all four tasks and four metrics (Accuracy, AUROC, F1 Macro, Cohen's Kappa) for all nine architectures. This directly visualizes the computational cost-to-benefit trade-off.

Together, the two figures now provide both absolute performance comparisons (Figure 1) and normalized efficiency comparisons (Figure 2), addressing the reviewer's concern comprehensively.

Comment 3: "The work presents just a passive observation without technical depth."

Stadtspital Zürich

Werner H. Spross
Stiftung zur Förderung
der Augenheilkunde

Stadtspital Zürich
Augenklinik
Birmensdorferstrasse 497, 8063 Zürich
stadtspital.ch

Sekretariat
T +41 44 416 42 42, F +41 44 416 42 44
augenklinik@stadtspital.ch

Chefarzt
Prof. Dr. med. Matthias Becker

We appreciate this concern and have deepened the analysis in several ways:

1. Parameter efficiency analysis (new Results subsection, Figure 2): We now quantitatively analyze performance per 100M parameters, revealing that compact models (particularly DINOv2-small) achieve efficiency up to 10× higher than RETFound variants, and that the relative ranking of models is remarkably consistent across all four metrics.
2. Expanded Discussion: We added a dedicated subsection on clinical translation considerations, discussing the trade-off between the 1.54 pp accuracy advantage of RETFound on DR and its 11x parameter cost, including absolute performance and operational impact (see Reviewer 2, Points 6 and 7 responses).
3. Expanded Limitations: We now explicitly address five limitation areas (external validation, interpretability, calibration, demographic fairness, computational efficiency) rather than the original four, acknowledging each as a direction for future work.
4. Hyperparameter fairness justification: We added a detailed explanation of the unified training protocol to ensure cross-model comparisons are valid (see Reviewer 2, Point 3 response).

We believe these additions transform the manuscript from a descriptive benchmark into a more analytically rigorous study.

Comment 4: "The paper discusses compact models for low resources environment however resources related metrics are not mentioned."

We acknowledge this important gap. To address this within our available data, we have:

1. Added a parameter efficiency analysis (Figure 2) that normalizes performance by model size (parameters per 100M), providing a principled proxy for computational cost.

Stadtspital Zürich
Augenklinik
Birmensdorferstrasse 497, 8063 Zürich
stadtspital.ch

Sekretariat
T +41 44 416 42 42, F +41 44 416 42 44
augenklinik@stadtspital.ch

Chefarzt
Prof. Dr. med. Matthias Becker

Parameter count scales approximately linearly with FLOPs and memory for transformer-based architectures within the same model family, making it a well-established proxy for deployment cost.

2. Explicitly acknowledged the absence of direct hardware profiling as a limitation in the revised Limitations section: *"The computational efficiency was not directly measured. We did not measure training time, inference latency, GPU memory consumption, or energy requirements across architectures... We discuss theoretical efficiency via parameter efficiency (accuracy per 100M parameters, Figure 2) but acknowledge that empirical measurements on representative hardware would strengthen the deployment guidance."*
3. Added a dedicated "Model efficiency" paragraph in the Discussion interpreting Figure 2 and its practical implications for resource-constrained deployment.

We believe the parameter efficiency analysis provides substantive, quantifiable evidence for the resource advantages of compact models, even in the absence of direct hardware profiling.

Comment 5: "The authors need to present in-depth analysis for a paper without novel architecture to be considered in high impact journals."

We agree and expect that the added analyses, parameter efficiency (Figure 2), the redesigned multi-panel Figure 1, clinical translation discussion, and the five-part expanded Limitations, collectively provide the analytical depth expected for an empirical benchmarking study. We also note that the controlled, multi-task, multi-modal design under unified training conditions is itself methodologically valuable, as it eliminates the confounds (different training protocols, different datasets, different hyperparameter tuning efforts) that limit the comparability of results reported across separate studies.

Stadtspital Zürich

Werner H. Spross Stiftung zur Förderung der Augenheilkunde

Stadtspital Zürich
Augenklinik
Birmensdorferstrasse 497, 8063 Zürich
stadtspital.ch

Sekretariat
T +41 44 416 42 42, F +41 44 416 42 44
augenklinik@stadtspital.ch

Chefarzt
Prof. Dr. med. Matthias Becker

Answers to Reviewer 2

Comment 1: "How did the authors address class imbalance during training, and can they provide per-class precision, recall, and F1-scores to better justify the observed advantage of RETFound?"

We thank the reviewer for this question. Regarding class imbalance handling we deliberately used unweighted cross-entropy loss without class reweighting, focal loss, or oversampling. This decision was made to isolate the effects of architecture and initialization strategy from task-specific loss engineering. Had we applied class-weighted or focal loss, performance differences between models could be attributed to loss function tuning rather than architectural or pretraining choices. We recognize that this may underestimate absolute performance on the DR task, particularly for the underrepresented severe classes, a point now discussed in our expanded Limitations section. Per-class metrics: We report macro-averaged precision, recall, and F1 across all classes to ensure fair cross-model comparison under class imbalance. This follows the reporting standard established by recent foundation model evaluations in retinal imaging, including the RETFound study itself (Zhou et al., Nature 2023), which reports aggregate rather than per-class metrics. Per-class metrics were not included as the focus of our study is the relative comparison of architectures and pretraining strategies at the aggregate level rather than absolute clinical performance on individual classes. We have further clarified it in the manuscript. We acknowledge that per-class analysis would be a valuable addition for clinical deployment studies and have noted this as a direction for future work in the Limitations.

Comment 2: "Were multiple-comparison corrections applied considering the large number of model-task comparisons?"

Stadtspital Zürich

Werner H. Spross
Stiftung zur Förderung
der Augenheilkunde

Stadtspital Zürich
Augenklinik
Birmensdorferstrasse 497, 8063 Zürich
stadtspital.ch

Sekretariat
T +41 44 416 42 42, F +41 44 416 42 44
augenklinik@stadtspital.ch

Chefarzt
Prof. Dr. med. Matthias Becker

We thank the reviewer for this important methodological point. We clarify two aspects:

Scope of statistical testing: The primary objective of our study is the empirical comparison of architectures and pretraining strategies. Model performance comparisons (Tables 4–5) are reported as point estimates on a single held-out validation set, following the convention of benchmarking studies. No inferential statistics (p-values) are computed for model-vs-model comparisons. The only statistical tests in our study are Mann–Whitney U tests comparing pretrained versus scratch-trained versions of each architecture - a secondary analysis addressing whether pretraining provides a statistically significant benefit. These comprise four tests (one per task: OCT, GL, DME, DR), with the following raw p-values:

Task	Raw p-value
OCT	0.003
GL	0.004
DME	0.022
DR	0.004

When applied both standard corrections:

- Benjamini–Hochberg FDR correction ($\alpha = 0.05$): All four comparisons remain statistically significant (adjusted p-values: 0.005, 0.005, 0.005, and 0.022, respectively). All adjusted p-values are below 0.05.
- Bonferroni correction ($\alpha/4 = 0.0125$): Three of four comparisons retain significance (OCT p=0.003, GL p=0.004, DR p=0.004). The DME comparison (p=0.022) does not meet this stricter threshold, although the effect size remains substantial (+10.70 percentage points).

Stadtspital Zürich
Augenklinik
Birmensdorferstrasse 497, 8063 Zürich
stadtspital.ch

Sekretariat
T +41 44 416 42 42, F +41 44 416 42 44
augenklinik@stadtspital.ch

Chefarzt
Prof. Dr. med. Matthias Becker

We note that the Benjamini–Hochberg procedure is widely considered more appropriate than Bonferroni for exploratory benchmarking studies, as it controls the expected proportion of false discoveries rather than the probability of any single false positive. Moreover, the consistency of the pretraining benefit, positive in direction across all four tasks and ranging from +5.18 to +18.41 percentage points, provides converging evidence that the effect is genuine rather than a statistical artifact. Given this does not represent the main scope of our manuscript, we have not added these statistical results to the manuscript itself.

Comment 3: "How was fairness ensured in terms of hyperparameter tuning, learning rates, training schedules, and data augmentation strategies across all models?"

We agree that fair comparison is essential when architectures differ substantially in size and inductive bias. We have strengthened the manuscript to make this explicit.
All models were trained under a unified protocol with the following held constant:

- Optimizer: AdamW
- Schedule: Cosine learning rate decay with 10% linear warmup
- Weight decay: 0.05; gradient clipping at max norm 1.0
- Effective batch size: 256 (via gradient accumulation)
- Epochs: 100, no early stopping, best validation accuracy reported
- Augmentation: Minimal (resize, center crop, normalization) — deliberately uniform across all models to isolate architecture effects
- Loss: Unweighted cross-entropy
- Hardware: Single NVIDIA RTX PRO 6000 GPU (Blackwell)
- Random seed: 42 for all experiments
- Precision: Mixed precision (bfloat16)

Stadtspital Zürich
Augenklinik
Birmensdorferstrasse 497, 8063 Zürich
stadtspital.ch

Sekretariat
T +41 44 416 42 42, F +41 44 416 42 44
augenklinik@stadtspital.ch

Chefarzt
Prof. Dr. med. Matthias Becker

Learning rates were the only hyperparameter varied per model, and were selected within ranges established in prior work for fine-tuning vision transformers (Steiner et al., 2022). Pretrained models received lower learning rates (2×10^{-4} to 1×10^{-3}) to preserve learned features, while scratch-trained models received higher rates (5×10^{-4} to 1×10^{-3}) following standard practice. The specific learning rate for each architecture is now listed in the revised Training Procedure section. We acknowledge that more extensive per-model hyperparameter search could shift absolute accuracies by 1–2 percentage points. However, given that the smallest reported between-model gap (1.54 pp on DR) approaches the typical magnitude of such tuning effects, our main conclusion - that compact models match large ones on three of four tasks – we believe is robust to this limitation.

Point 4: "Can the authors provide training time, inference latency, FLOPs, GPU memory consumption, and energy requirements for each model?"

We share the reviewer's view that direct hardware profiling would strengthen the deployment-cost argument. Unfortunately, we do not have this data. We have addressed this in two ways:

1. Added a parameter efficiency analysis (Figure 2) that quantifies performance per 100M parameters across all architectures, tasks, and metrics. Parameter count serves as a well-established proxy for computational cost, as it scales approximately with FLOPs and memory for transformer-based architectures within the same model family. Figure 2 shows that DINOv2-small (22.8M) achieves efficiency values up to 10× higher than the 303M RETFound variants, with the relative ranking consistent across all four metrics.
2. Explicitly acknowledged the absence of direct hardware measurements as a limitation in the revised Limitations section, noting that empirical measurements on representative hardware would further strengthen deployment guidance.

Stadtspital Zürich

Werner H. Spross
Stiftung zur Förderung
der Augenheilkunde

Stadtspital Zürich
Augenklinik
Birmensdorferstrasse 497, 8063 Zürich
stadtspital.ch

Sekretariat
T +41 44 416 42 42, F +41 44 416 42 44
augenklinik@stadtspital.ch

Chefarzt
Prof. Dr. med. Matthias Becker

We believe the parameter efficiency analysis provides substantive, quantifiable evidence for the computational advantages of compact models and respectfully suggest that direct hardware profiling, while desirable, is not essential to the core conclusions of the study.

Point 5: "Have the authors evaluated the models on external datasets or cross-domain settings to verify the robustness and generalizability?"

No, as all models were evaluated on a single dataset (or dataset combination) per task. We have not performed external validation on independent cohorts. We fully agree that external validation is essential before clinical deployment conclusions can be drawn.
This is now explicitly addressed as the first item in our expanded Limitations section: *"First there is no external validation cohort. Each task was evaluated on a single dataset or dataset combination (OCT C8, IDRiD+Messidor-2, AIROGS+PAPILA, EAM). Generalizability to other data sources, imaging devices, geographic populations, and acquisition protocols was not assessed."* We have also added a statement in the Discussion noting that *"benchmark results should not be extrapolated to real-world screening contexts without further validation"* and that *"external validation studies on independent cohorts would be required before drawing clinical conclusions."* We view external validation as the most important next step for translating our findings and have framed it accordingly.

Point 6: "Could the authors include feature visualization, attention maps, or representation similarity analyses to explain why RETFound performs better on DR severity grading?"

We agree that mechanistic understanding of RETFound's DR advantage would be valuable. We did not include feature visualization or attention map analysis in this study, as it falls outside the

Stadtspital Zürich
Augenklinik
Birmensdorferstrasse 497, 8063 Zürich
stadtspital.ch

Sekretariat
T +41 44 416 42 42, F +41 44 416 42 44
augenklinik@stadtspital.ch

Chefarzt
Prof. Dr. med. Matthias Becker

scope of our controlled benchmarking design (which focuses on performance comparisons rather than interpretability). However, we have added a paragraph in the Discussion outlining this direction and proposing a testable hypothesis: *"Mechanistic understanding of RETFound's DR advantage may benefit from future work using attention rollout, Grad-CAM, or representation similarity analysis comparing feature spaces of compact models and RETFound on DR images. We hypothesize that RETFound's retinal-specific pretraining yields representations more sensitive to fine pathological features (microaneurysms, small hemorrhages, IRMA) that are diagnostic of severe DR but underrepresented in ImageNet. Testing this hypothesis is an important direction for follow-up work."* Additionally, we have added the absence of interpretability analysis as an explicit limitation: *"Second, no feature visualization or mechanistic interpretability analysis was performed... Such analyses are necessary to establish mechanistic understanding of when and why domain-specific pretraining helps."*

Point 7: "Given that the performance difference is only 1.54 percentage points, is this improvement clinically meaningful?"

We thank the reviewer for raising this crucial question. We have expanded the Discussion to contextualize the clinical trade-off across multiple dimensions:
Absolute performance: All models on DR remain substantially below ceiling (best accuracy 71.15%), reflecting the inherent difficulty of 5-class ordinal grading under severe class imbalance. The accuracy gap of 1.54 pp corresponds to a relatively small improvement in Cohen's Kappa, which is below the threshold typically considered "clinically meaningful" in published guidelines, though formal thresholds depend on the specific application.
Operational impact: For every 1,000 screened patients at a base rate of ~5% referable DR, the 1.54 pp accuracy gain corresponds to approximately 15 additional correctly classified patients.

Stadtspital Zürich

Werner H. Spross Stiftung zur Förderung der Augenheilkunde

Stadtspital Zürich
Augenklinik
Birmensdorferstrasse 497, 8063 Zürich
stadtspital.ch

Sekretariat
T +41 44 416 42 42, F +41 44 416 42 44
augenklinik@stadtspital.ch

Chefarzt
Prof. Dr. med. Matthias Becker

Whether this justifies the 11x increase in parameter count depends on the screening context: in high-throughput population screening, the cumulative computational overhead may be prohibitive; in low-volume specialist referral settings, the marginal gain may be worthwhile.
Parameter efficiency: Figure 2 shows that RETFound's per-parameter efficiency on DR is approximately an order of magnitude lower than compact models, meaning the marginal accuracy gain comes at a disproportionately high computational cost.
We conclude that the clinical meaningfulness of RETFound's advantage depends on the deployment context and clinical priorities (sensitivity vs. specificity, throughput vs. accuracy), and we have framed our recommendation accordingly: *"Large domain-specific foundation models are better justified when the task involves severity grading with skewed class distributions"* — rather than universally recommended.

Point 8: "Include the current related work"

We thank the reviewer for bringing these references to our attention. We have reviewed all five suggested publications. Upon examination, we note that several of these works share common authorship, which could introduce citation bias if all were included. As such we have refrained from adding them.

Point 9: "Please provide the URLs or access links for all datasets used in the study."

We have added direct URLs for all datasets in the revised Methods section:

- OCT (Retinal OCT C8): https://www.kaggle.com/datasets/obulisainaren/retinal-oct-c8
- DME (IDRiD): https://idrid.grand-challenge.org/Data/
- DME (Messidor-2): https://www.kaggle.com/datasets/mariaherrerot/messidor2preprocess

Stadtspital Zürich

Werner H. Spross
Stiftung zur Förderung
der Augenheilkunde

Stadtspital Zürich
Augenklinik
Birmensdorferstrasse 497, 8063 Zürich
stadtspital.ch

Sekretariat
T +41 44 416 42 42, F +41 44 416 42 44
augenklinik@stadtspital.ch

Chefarzt
Prof. Dr. med. Matthias Becker

- GL (AIROGS): https://airogs.grand-challenge.org
- GL (PAPILA): https://www.kaggle.com/datasets/orvile/papila-retinal-fundus-images
- DR (EAM): https://www.kaggle.com/datasets/ascanipek/eyepacs-aptos-messidor-diabetic-retinopathy

All datasets are open-access, and access details are now provided alongside the dataset descriptions in the Methods.

We trust that these revisions adequately address all reviewer and journal concerns and welcome any further feedback.

Sincerely,

Rui Santos, on behalf of all co-authors:
Dávid Isztl, Tahm Spitznagel and Gábor Márk Somfai
Stadtspital Zürich, Department of Ophthalmology Spross Research Institute, Zürich, Switzerland
