# Supplementary material for: Compact vision models match domain-specific foundation models for several retinal imaging classification tasks: A systematic benchmark
Source: PLoS One. 2026 Aug 18;21(8):e0356202. doi: 10.1371/journal.pone.0356202 (PMC13484977; doi:10.1371/journal.pone.0356202)
Supplement: Peer review history

# Peer review: Decision Letter 0

**DOI:** 10.1371/journal.pone.0356202.r001

**Authors:** Xin Sun

16 Jun 2026

Dear Dr. Santos,

Thank you for submitting your manuscript to PLOS ONE. After careful consideration, we feel that it has merit but does not fully meet PLOS ONE’s publication criteria as it currently stands. Therefore, we invite you to submit a revised version of the manuscript that addresses the points raised during the review process.

Your manuscript has been evaluated by two experts of the field and several scientific concerns have been raised. Please address them one by one in your revision.

Please submit your revised manuscript by Jul 31 2026 11:59PM. If you will need more time than this to complete your revisions, please reply to this message or contact the journal office at plosone@plos.org. When you're ready to submit your revision, log on to https://www.editorialmanager.com/pone/ and select the 'Submissions Needing Revision' folder to locate your manuscript file.

- A letter that responds to each point raised by the academic editor and reviewer(s). You should upload this letter as a separate file labeled 'Response to Reviewers'.
- A marked-up copy of your manuscript that highlights changes made to the original version. You should upload this as a separate file labeled 'Revised Manuscript with Track Changes'.
- An unmarked version of your revised paper without tracked changes. You should upload this as a separate file labeled 'Manuscript'.

If you would like to make changes to your financial disclosure, please include your updated statement in your cover letter. Guidelines for resubmitting your figure files are available below the reviewer comments at the end of this letter.

If applicable, we recommend that you deposit your laboratory protocols in protocols.io to enhance the reproducibility of your results. Protocols.io assigns your protocol its own identifier (DOI) so that it can be cited independently in the future. For instructions see: https://journals.plos.org/plosone/s/submission-guidelines#loc-laboratory-protocols. Additionally, PLOS ONE offers an option for publishing peer-reviewed Lab Protocol articles, which describe protocols hosted on protocols.io. Read more information on sharing protocols at https://plos.org/protocols?utm_medium=editorial-email&utm_source=authorletters&utm_campaign=protocols.

As the corresponding author, your ORCID iD is verified in the submission system and will appear in the published article. PLOS supports the use of ORCID, and we encourage all coauthors to register for an ORCID iD and use it as well. Please encourage your coauthors to verify their ORCID iD within the submission system before final acceptance, as unverified ORCID iDs will not appear in the published article. *Only*  the individual author can complete the verification step; PLOS staff *cannot*  verify ORCID iDs on behalf of authors.

We look forward to receiving your revised manuscript.

Kind regards,

Xin Sun, PhD

Staff Editor

PLOS One

Journal Requirements:

When submitting your revision, we need you to address these additional requirements.

1. Please ensure that your manuscript meets PLOS ONE's style requirements, including those for file naming. The PLOS ONE style templates can be found at

https://journals.plos.org/plosone/s/file?id=wjVg/PLOSOne_formatting_sample_main_body.pdf and https://journals.plos.org/plosone/s/file?id=ba62/PLOSOne_formatting_sample_title_authors_affiliations.pdf

2. Please note that PLOS One has specific guidelines on code sharing for submissions in which author-generated code underpins the findings in the manuscript. In these cases, all author-generated code must be made available without restrictions upon publication of the work. Please review our guidelines at https://journals.plos.org/plosone/s/materials-and-software-sharing#loc-sharing-code and ensure that your code is shared in a way that follows best practice and facilitates reproducibility and reuse.

3. Your ethics statement should only appear in the Methods section of your manuscript. If your ethics statement is written in any section besides the Methods, please delete it from any other section.

4. Thank you for stating the following financial disclosure:

The work was supported by the Werner H. Spross Stiftung zur Förderung der Augenheilkunde

Please state what role the funders took in the study. If the funders had no role, please state: "The funders had no role in study design, data collection and analysis, decision to publish, or preparation of the manuscript."

If this statement is not correct you must amend it as needed.

Please include this amended Role of Funder statement in your cover letter; we will change the online submission form on your behalf.

5. If the reviewer comments include a recommendation to cite specific previously published works, please review and evaluate these publications to determine whether they are relevant and should be cited. There is no requirement to cite these works unless the editor has indicated otherwise.

[Note: HTML markup is below. Please do not edit.]

Reviewers' comments:

Reviewer's Responses to Questions

**Comments to the Author**

1. Is the manuscript technically sound, and do the data support the conclusions?

Reviewer #1: Yes

Reviewer #2: Yes

**********

2. Has the statistical analysis been performed appropriately and rigorously?

Reviewer #1: Yes

Reviewer #2: Yes

**********

3. Have the authors made all data underlying the findings in their manuscript fully available?

The PLOS Data policy

Reviewer #1: Yes

Reviewer #2: Yes

**********

4. Is the manuscript presented in an intelligible fashion and written in standard English?

Reviewer #1: Yes

Reviewer #2: Yes

**********

Reviewer #1: =The study investigates whether massive, domain-specific medical foundation models genuinely outperform much smaller, general-purpose computer vision architectures when applied to routine retinal imaging classification tasks.

-no architecture novelty (the authors propose no new framework).

- there are not enough figures to show the difference between models' performance.

- the work presents just a passive observation without technical depth.

- the paper discusses compact models for low resources environment however resources related metrics are not mentioned.

the authors need to present in-depth analysis for a paper without novel architecture to be considered in high impact journals

Reviewer #2: 1. The manuscript reports superior performance of RETFound on DR severity grading, where the most severe class represents only 8% of the dataset. How did the authors address class imbalance during training, and can they provide per-class precision, recall, and F1-scores to better justify the observed advantage of RETFound?

2. The authors employed the Mann–Whitney U test to compare pretrained and randomly initialized models. Were multiple-comparison corrections (e.g., Bonferroni or Benjamini–Hochberg adjustment) applied considering the large number of model-task comparisons? Please clarify. 3.

3. Since the evaluated models differ substantially in architecture and parameter count (22.8M–303M parameters), how was fairness ensured in terms of hyperparameter tuning, learning rates, training schedules, and data augmentation strategies across all models? 4.

4. While compact models achieved comparable performance to larger foundation models, the manuscript does not discuss computational efficiency. Can the authors provide training time, inference latency, FLOPs, GPU memory consumption, and energy requirements for each model to support the practical advantages of compact architectures? 4.

5. The conclusions suggest that compact general-purpose models are sufficient for most retinal classification tasks. Have the authors evaluated the models on external datasets or cross-domain settings to verify the robustness and generalizability of this conclusion? 5.

6. What evidence supports the claim that domain-specific foundation models provide additional value primarily for severity grading tasks? Could the authors include feature visualization, attention maps, or representation similarity analyses to explain why RETFound performs better on DR severity grading? 6.

7. Given that the performance difference between RETFound and the best compact model on DR grading is only 1.54 percentage points, is this improvement clinically meaningful? Please provide a discussion on the trade-off between accuracy gain, model size, computational cost, and real-world deployment feasibility in ophthalmology settings.

8. Include the current related work: Detection of glaucoma in retinal fundus images using fast fuzzy C means clustering approach , Introduction to artificial intelligence and current trends, A three-stage novel framework for efficient and automatic glaucoma classification from retinal fundus images, An analytical study on machine learning techniques, Histogram of oriented gradients (HOG)-based artificial neural network (ANN) classifier for glaucoma detection

9. Please provide the URLs or access links for all datasets used in the study, along with appropriate citations and access details, to facilitate verification, reproducibility, and future research by readers.

**********

what does this mean?). If published, this will include your full peer review and any attached files.

If you choose “no”, your identity will remain anonymous but your review may still be made public.

**Do you want your identity to be public for this peer review?**  For information about this choice, including consent withdrawal, please see our Privacy Policy

Reviewer #1: No

Reviewer #2: No

**********

[NOTE: If reviewer comments were submitted as an attachment file, they will be attached to this email and accessible via the submission site. Please log into your account, locate the manuscript record, and check for the action link "View Attachments". If this link does not appear, there are no attachment files.]

To ensure your figures meet our technical requirements, please review our figure guidelines: https://journals.plos.org/plosone/s/figures

You may also use PLOS’s free figure tool, NAAS, to help you prepare publication quality figures: https://journals.plos.org/plosone/s/figures#loc-tools-for-figure-preparation.

NAAS will assess whether your figures meet our technical requirements by comparing each figure against our figure specifications.

# Author comment: Author response to Decision Letter 1

**DOI:** 10.1371/journal.pone.0356202.r002

30 Jun 2026

25th June 2026

To the editor Xin Sun (PhD)

PLOS ONE

Public Library of Science

Rebuttal Letter to reviewers for manuscript: "Compact vision models match domain-specific foundation models for several retinal imaging classification tasks: A systematic benchmark"

We thank the editor and reviewers for their thoughtful and constructive comments, which have substantially strengthened the manuscript. Below, we address each point in detail.

All changes are highlighted in the marked-up manuscript.

Journal Requirements

Requirement 1: PLOS ONE style requirements. We have reviewed and confirmed that the manuscript conforms to PLOS ONE formatting guidelines, including file naming conventions, section structure, and reference formatting.

Requirement 2: We have added a "Data availability" statement to the manuscript specifying dataset access links, and code will be deposited at the time of acceptance.

Requirement 3: Ethics statement location. We confirm that the ethics statement has been moved to the Methods section as a final subsection ("This study used exclusively publicly available, de-identified datasets. No ethics approval was required.") and has been removed from any other section.

Requirement 4: Funder role statement. We have updated the Funding section to read: "This work was supported by the Werner H. Spross-Stiftung. The funders had no role in study design, data collection and analysis, decision to publish, or preparation of the manuscript."

Requirement 5: Reviewer-recommended citations. We have reviewed the publications suggested by Reviewer 2 (Point 8) and have addressed this in our response below (see Reviewer 2, Point 8).

Answers to Reviewer 1

Comment 1: "No architecture novelty (the authors propose no new framework)."

We thank the reviewer for this comment and agree that this study does not propose a novel architecture. We respectfully clarify that our manuscript is an empirical benchmarking study, not a methods or architecture-development paper. Its scientific contribution lies in providing the first controlled, multi-task, multi-modal comparison of compact general-purpose models against domain-specific foundation models (RETFound) under identical training conditions. This is a distinct and valuable contribution, as the field currently lacks systematic evidence about when domain-specific scale is justified versus when compact, off-the-shelf models suffice. We have added an explicit statement in the Introduction clarifying this scope:

"Our contribution is empirical rather than architectural: we provide a controlled comparison of compact and domain-specific foundation models under identical training conditions across multiple tasks and modalities, as a reference for practitioners choosing between compact general-purpose models and larger domain-specific alternatives for the future development of vision-based Foundation Models.”

We believe the value of systematic benchmarking is well-established in the machine learning community. Our study serves the same purpose for retinal imaging specifically.

Comment 2: "There are not enough figures to show the difference between models' performance."

We agree that the original single-figure submission was insufficient. We have substantially expanded the visual presentation:

• Figure 1 has been completely redesigned. It now comprises a multi-panel figure showing validation Accuracy, AUROC, and F1 Macro for all four tasks (OCT, DME, GL, DR) plotted against model size, with pretrained and scratch-trained models shown separately, Pareto-optimal models highlighted with annotated markers, and per-subplot linear regression lines with r² values. This provides a comprehensive visual comparison across all models, tasks, and metrics simultaneously.

• Figure 2 is newly added, presenting a 4-panel bar chart showing parameter efficiency (performance per 100M parameters) across all four tasks and four metrics (Accuracy, AUROC, F1 Macro, Cohen's Kappa) for all nine architectures. This directly visualizes the computational cost-to-benefit trade-off.

Together, the two figures now provide both absolute performance comparisons (Figure 1) and normalized efficiency comparisons (Figure 2), addressing the reviewer's concern comprehensively.

Comment 3: "The work presents just a passive observation without technical depth."

We appreciate this concern and have deepened the analysis in several ways:

1. Parameter efficiency analysis (new Results subsection, Figure 2): We now quantitatively analyze performance per 100M parameters, revealing that compact models (particularly DINOv2-small) achieve efficiency up to 10× higher than RETFound variants, and that the relative ranking of models is remarkably consistent across all four metrics.

2. Expanded Discussion: We added a dedicated subsection on clinical translation considerations, discussing the trade-off between the 1.54 pp accuracy advantage of RETFound on DR and its 11x parameter cost, including absolute performance and operational impact (see Reviewer 2, Points 6 and 7 responses).

3. Expanded Limitations: We now explicitly address five limitation areas (external validation, interpretability, calibration, demographic fairness, computational efficiency) rather than the original four, acknowledging each as a direction for future work.

4. Hyperparameter fairness justification: We added a detailed explanation of the unified training protocol to ensure cross-model comparisons are valid (see Reviewer 2, Point 3 response).

We believe these additions transform the manuscript from a descriptive benchmark into a more analytically rigorous study.

Comment 4: "The paper discusses compact models for low resources environment however resources related metrics are not mentioned."

We acknowledge this important gap. To address this within our available data, we have:

1. Added a parameter efficiency analysis (Figure 2) that normalizes performance by model size (parameters per 100M), providing a principled proxy for computational cost. Parameter count scales approximately linearly with FLOPs and memory for transformer-based architectures within the same model family, making it a well-established proxy for deployment cost.

2. Explicitly acknowledged the absence of direct hardware profiling as a limitation in the revised Limitations section: "The computational efficiency was not directly measured. We did not measure training time, inference latency, GPU memory consumption, or energy requirements across architectures... We discuss theoretical efficiency via parameter efficiency (accuracy per 100M parameters, Figure 2) but acknowledge that empirical measurements on representative hardware would strengthen the deployment guidance."

3. Added a dedicated "Model efficiency" paragraph in the Discussion interpreting Figure 2 and its practical implications for resource-constrained deployment.

We believe the parameter efficiency analysis provides substantive, quantifiable evidence for the resource advantages of compact models, even in the absence of direct hardware profiling.

Comment 5: "The authors need to present in-depth analysis for a paper without novel architecture to be considered in high impact journals."

We agree and expect that the added analyses, parameter efficiency (Figure 2), the redesigned multi-panel Figure 1, clinical translation discussion, and the five-part expanded Limitations, collectively provide the analytical depth expected for an empirical benchmarking study. We also note that the controlled, multi-task, multi-modal design under unified training conditions is itself methodologically valuable, as it eliminates the confounds (different training protocols, different datasets, different hyperparameter tuning efforts) that limit the comparability of results reported across separate studies.

Answers to Reviewer 2

Comment 1: "How did the authors address class imbalance during training, and can they provide per-class precision, recall, and F1-scores to better justify the observed advantage of RETFound?"

We thank the reviewer for this question. Regarding class imbalance handling we deliberately used unweighted cross-entropy loss without class reweighting, focal loss, or oversampling. This decision was made to isolate the effects of architecture and initialization strategy from task-specific loss engineering. Had we applied class-weighted or focal loss, performance differences between models could be attributed to loss function tuning rather than architectural or pretraining choices. We recognize that this may underestimate absolute performance on the DR task, particularly for the underrepresented severe classes, a point now discussed in our expanded Limitations section. Per-class metrics: We report macro-averaged precision, recall, and F1 across all classes to ensure fair cross-model comparison under class imbalance. This follows the reporting standard established by recent foundation model evaluations in retinal imaging, including the RETFound study itself (Zhou et al., Nature 2023), which reports aggregate rather than per-class metrics. Per-class metrics were not included as the focus of our study is the relative comparison of architectures and pretraining strategies at the aggregate level rather than absolute clinical performance on individual classes. We have further clarified it in the manuscript. We acknowledge that per-class analysis would be a valuable addition for clinical deployment studies and have noted this as a direction for future work in the Limitations.

Comment 2: "Were multiple-comparison corrections applied considering the large number of model-task comparisons?"

We thank the reviewer for this important methodological point. We clarify two aspects:

Scope of statistical testing: The primary objective of our study is the empirical comparison of architectures and pretraining strategies. Model performance comparisons (Tables 4–5) are reported as point estimates on a single held-out validation set, following the convention of benchmarking studies. No inferential statistics (p-values) are computed for model-vs-model comparisons. The only statistical tests in our study are Mann–Whitney U tests comparing pretrained versus scratch-trained versions of each architecture - a secondary analysis addressing whether pretraining provides a statistically significant benefit. These comprise four tests (one per task: OCT, GL, DME, DR), with the following raw p-values:

Task Raw p-value

OCT 0.003

GL 0.004

DME 0.022

DR 0.004

When applied both standard corrections:

• Benjamini–Hochberg FDR correction (α = 0.05): All four comparisons remain statistically significant (adjusted p-values: 0.005, 0.005, 0.005, and 0.022, respectively). All adjusted p-values are below 0.05.

• Bonferroni correction (α/4 = 0.0125): Three of four comparisons retain significance (OCT p=0.003, GL p=0.004, DR p=0.004). The DME comparison (p=0.022) does not meet this stricter threshold, although the effect size remains substantial (+10.70 percentage points).

We note that the Benjamini–Hochberg procedure is widely considered more appropriate than Bonferroni for exploratory benchmarking studies, as it controls the expected proportion of false discoveries rather than the probability of any single false positive. Moreover, the consistency of the pretraining benefit, positive in direction across all four tasks and ranging from +5.18 to +18.41 percentage points, provides converging evidence that the effect is genuine rather than a statistical artifact. Given this does not represent the main scope of our manuscript, we have not added these statistical results to the manuscript itself.

Comment 3: "How was fairness ensured in terms of hyperparameter tuning, learning rates, training schedules, and data augmentation strategies across all models?"

We agree that fair comparison is essential when architectures differ substantially in size and inductive bias. We have strengthened the manuscript to make this explicit.

All models were trained under a unified protocol with the following held constant:

• Optimizer: AdamW

• Schedule: Cosine learning rate decay with 10% linear warmup

• Weight decay: 0.05; gradient clipping at max norm 1.0

• Effective batch size: 256 (via gradient accumulation)

• Epochs: 100, no early stopping, best validation accuracy reported

• Augmentation: Minimal (resize, center crop, normalization) — deliberately uniform across all models to isolate architecture effects

• Loss: Unweighted cross-entropy

• Hardware: Single NVIDIA RTX PRO 6000 GPU (Blackwell)

• Random seed: 42 for all experiments

• Precision: Mixed precision (bfloat16)

Learning rates were the only hyperparameter varied per model, and were selected within ranges established in prior work for fine-tuning vision transformers (Steiner et al., 2022). Pretrained models received lower learning rates (2×10⁻⁴ to 1×10⁻³) to preserve learned features, while scratch-trained models received higher rates (5×10⁻⁴ to 1×10⁻³) following standard practice. The specific learning rate for each architecture is now listed in the revised Training Procedure section.

We acknowledge that more extensive per-model hyperparameter search could shift absolute accuracies by 1–2 percentage points. However, given that the smallest reported between-model gap (1.54 pp on DR) approaches the typical magnitude of such tuning effects, our main conclusion - that compact models match large ones on three of four tasks – we believe is robust to this limitation.

Point 4: "Can the authors provide training time, inference latency, FLOPs, GPU memory consumption, and energy requirements for each model?"

We share the reviewer's view that direct hardware profiling would strengthen the deployment-cost argument. Unfortunately, we do not have this data. We have addressed this in two ways:

1. Added a parameter efficiency analysis (Figure 2) that quantifies performance per 100M parameters across all architectures, tasks, and metrics. Parameter count serves as a well-established proxy for computational cost, as it scales approximately with FLOPs and memory for transformer-based architectures within the same model family. Figure 2 shows that DINOv2-small (22.8M) achieves efficiency values up to 10× higher than the 303M RETFound variants, with the relative ranking consistent across all four metrics.

2. Explicitly acknowledged the absence of direct hardware measurements as a limitation in the revised Limitations section, noting that empirical measurements on representative hardware would further strengthen deployment guidance.

We believe the parameter efficiency analysis provides substantive, quantifiable evidence for the computational advantages of compact models and respectfully suggest that direct hardware profiling, while desirable, is not essential to the core conclusions of the study.

Point 5: "Have the authors evaluated the models on external datasets or cross-domain settings to verify the robustness and generalizability?"

No, as all models were evaluated on a single dataset (or dataset combination) per task. We have not performed external validation on independent cohorts. We fully agree that external validation is essential before clinical deployment conclusions can be drawn.

This is now explicitly addressed as the first item in our expanded Limitations section: "First there is no external validation cohort. Each task was evaluated on a single dataset or dataset combination (OCT C8, IDRiD+Messidor-2, AIROGS+PAPILA, EAM). Generalizability to other data sources, imaging devices, geographic populations, and acquisition protocols was not assessed."

We have also added a statement in the Discussion noting that "benchmark results should not be extrapolated to real-world screening contexts without further validation" and that "external validation studies on independent cohorts would be required before drawing clinical conclusions." We view external validation as the most important next step for translating our findings and have framed it accordingly.

Point 6: "Could the authors include feature visualization, attention maps, or representation similarity analyses to explain why RETFound performs better on DR severity grading?"

We agree that mechanistic understanding of RETFound's DR advantage would be valuable

Attachment Submitted filename: rebuttal letter.pdf

# Peer review: Decision Letter 1

**DOI:** 10.1371/journal.pone.0356202.r003

**Authors:** Jia-Lang Xu

30 Jul 2026

Compact vision models match domain-specific foundation models for several retinal imaging classification tasks: A systematic benchmark

PONE-D-26-18392R1

Dear Dr. Santos,

We’re pleased to inform you that your manuscript has been judged scientifically suitable for publication and will be formally accepted for publication once it meets all outstanding technical requirements.

Within one week, you’ll receive an e-mail detailing the required amendments. When these have been addressed, you’ll receive a formal acceptance letter and your manuscript will be scheduled for publication.

An invoice will be generated when your article is formally accepted. Please note, if your institution has a publishing partnership with PLOS and your article meets the relevant criteria, all or part of your publication costs will be covered. Please make sure your user information is up-to-date by logging into Editorial Manager at Editorial Manager® and clicking the ‘Update My Information' link at the top of the page. For questions related to billing, please contact billing support.

If your institution or institutions have a press office, please notify them about your upcoming paper to help maximize its impact. If they’ll be preparing press materials, please inform our press team as soon as possible -- no later than 48 hours after receiving the formal acceptance. Your manuscript will remain under strict press embargo until 2 pm Eastern Time on the date of publication. For more information, please contact onepress@plos.org.

Kind regards,

Jia-Lang Xu

Academic Editor

PLOS One

Additional Editor Comments (optional):

During the review process, two reviewers provided recommendations for acceptance. Based on their positive evaluations, the manuscript is recommended for acceptance.

Reviewers' comments:

Reviewer's Responses to Questions

**Comments to the Author**

Reviewer #1: All comments have been addressed

Reviewer #2: All comments have been addressed

**********

2. Is the manuscript technically sound, and do the data support the conclusions?

Reviewer #1: Yes

Reviewer #2: Partly

**********

3. Has the statistical analysis been performed appropriately and rigorously?

Reviewer #1: N/A

Reviewer #2: Yes

**********

4. Have the authors made all data underlying the findings in their manuscript fully available?

The PLOS Data policy

Reviewer #1: Yes

Reviewer #2: Yes

**********

5. Is the manuscript presented in an intelligible fashion and written in standard English?

Reviewer #1: Yes

Reviewer #2: Yes

**********

Reviewer #1: I want to thank authors for answering my comments and questions. All my comments were answered correctly

Reviewer #2: The authors have incorporated all the suggested revisions; therefore, the manuscript can be accepted for publication.

**********

what does this mean?). If published, this will include your full peer review and any attached files.

If you choose “no”, your identity will remain anonymous but your review may still be made public.

**Do you want your identity to be public for this peer review?**  For information about this choice, including consent withdrawal, please see our Privacy Policy

Reviewer #1: **Yes:** Ayman Youssef

Reviewer #2: No

**********

# Editor report: Acceptance letter

**DOI:** 10.1371/journal.pone.0356202.r004

**Authors:** Jia-Lang Xu

PONE-D-26-18392R1

PLOS One

Dear Dr. Santos,

I'm pleased to inform you that your manuscript has been deemed suitable for publication in PLOS One. Congratulations! Your manuscript is now being handed over to our production team.

At this stage, our production department will prepare your paper for publication. This includes ensuring the following:

* All references, tables, and figures are properly cited

* All relevant supporting information is included in the manuscript submission,

* There are no issues that prevent the paper from being properly typeset

You will receive further instructions from the production team, including instructions on how to review your proof when it is ready. Please keep in mind that we are working through a large volume of accepted articles, so please give us a few days to review your paper and let you know the next and final steps.

Lastly, if your institution or institutions have a press office, please let them know about your upcoming paper now to help maximize its impact. If they'll be preparing press materials, please inform our press team within the next 48 hours. Your manuscript will remain under strict press embargo until 2 pm Eastern Time on the date of publication. For more information, please contact onepress@plos.org.

You will receive an invoice from PLOS for your publication fee after your manuscript has reached the completed accept phase. If you receive an email requesting payment before acceptance or for any other service, this may be a phishing scheme. Learn how to identify phishing emails and protect your accounts at https://explore.plos.org/phishing.

If we can help with anything else, please email us at customercare@plos.org.

Thank you for submitting your work to PLOS ONE and supporting open access.

Kind regards,

PLOS ONE Editorial Office Staff

on behalf of

Dr. Jia-Lang Xu

Academic Editor

PLOS One
